# Efficacy of Platelet‐Rich Plasma Injections Versus Corticosteroid Injections in the Management of Chronic Low Back Pain Secondary to Sacroiliitis

**DOI:** 10.1155/prm/8179046

**Published:** 2026-06-22

**Authors:** Karen Dayana Saavedra Pérez, Nicole Andrea Bonilla Silva, Sofía Martínez Neader, Lina Andrea Gómez

**Affiliations:** ^1^ Universidad de La Sabana, Chía, Colombia, unisabana.edu.co; ^2^ Department of Biosciences, Biomedical Research Center (CIBUS), Faculty of Medicine, Universidad de La Sabana, Chía, Colombia, unisabana.edu.co

## Abstract

**Objectives:**

Low back pain secondary to sacroiliitis (SI) significantly affects patients’ quality of life. Although corticosteroid injections are commonly used, their effects may be temporary and have adverse effects. Platelet‐rich plasma (PRP) has emerged as a potential alternative; however, comparative evidence remains limited. Therefore, this study aimed to compare the efficacy of PRP versus corticosteroid injections in managing chronic low back pain secondary to SI to provide evidence to guide clinical decision‐making and optimize treatment strategies across different clinical contexts.

**Methods:**

This systematic review was conducted according to the Preferred Reporting Items for Systematic Reviews and Meta‐Analyses (PRISMA) guidelines. A total of 265 articles were identified in the databases, of which 4 met the defined inclusion and exclusion criteria. The search window was set to 2020–2025. The search window was limited to the past four years to capture recent and relevant evidence, given the field’s rapid evolution.

**Results:**

Based on the selected articles, corticosteroids offer short‐term relief, while PRP shows greater and longer‐lasting benefits. Additionally, PRP was associated with a lower need for additional interventions and rescue analgesia, demonstrating a sustained effect over time.

**Conclusions:**

Corticosteroids provide faster relief within 2–4 weeks, while PRP offers longer‐lasting benefits from the eighth week up to 6 months. PRP also reduces the need for additional interventions and has no significant adverse effects, unlike corticosteroids. These findings suggest PRP as a safer long‐term alternative for patients needing repeated or prolonged treatment. Future research should implement studies with a larger number of patients, longer observation periods, and follow‐up to fully understand the benefits and limitations of both therapies.

## 1. Introduction

Low back pain is the most prevalent musculoskeletal disease worldwide. It is recognized as the leading cause of disability globally, with a lifetime prevalence of 84% in the adult population, resulting in a significant burden both for those who suffer from it and for healthcare systems [1, 2].

Chronic low back pain can have various etiologies, such as disc injuries, degeneration, trauma, osteoporosis, spondyloarthropathies, and sacroiliitis (SI). SI reaches up to 25% prevalence in patients with low back pain [3, 4]. This condition corresponds to the inflammation of one or both sacroiliac joints and can result in severe disability and a significant decrease in patients′ quality of life [5, 6]. SI, recognized as one of the main etiologies of low back pain, results from substantial mechanical stress caused by a combination of axial load and sudden rotation [7, 8]. Sacroiliac joint dysfunction has different etiologies, such as inflammatory types corresponding to spondyloarthropathies and rheumatoid arthritis, traumatic types resulting from direct blows or repetitive stress on the joint; likewise, this localized pain can be generated by degenerative changes associated with age [9]. It is common for patients suffering from SI to have an incorrect or nonspecific diagnosis despite the available diagnostic methods [10]. Therefore, this disease is often underdiagnosed, and ineffective or inappropriate treatments are provided [11].

In Latin America, chronic low back pain has a major impact on the working population, creating significant social and economic consequences. In Mexico, prevalence among workers has been reported between 12% and 26%, in Argentina, it is one of the leading causes of disability affecting labor productivity, and in Brazil, it has resulted in thousands of permanent disability pensions. This regional burden highlights the urgent need for cost‐effective treatment alternatives that can improve quality of life and reduce disability [12–16].

The treatment of sacroiliac joint pain as a cause of chronic low back pain remains a therapeutic challenge, as current alternatives such as systemic therapies, including medications, physiotherapy, radiofrequency ablation, and joint fusion, do not completely relieve pain [17, 18]. Corticosteroid injections represent one of the most widely performed interventions for pain management worldwide. Their primary purpose is to reduce inflammation and alleviate pain, offering patients rapid symptomatic relief [19].

The way these drugs act is based on two mechanisms of action: they can passively diffuse through cell membranes to subsequently bind to soluble cytoplasmic receptors; this hormone–receptor complex moves to the nucleus and regulates the transcription of a limited number of target genes, or they can modulate the second messenger cascade through nongenomic pathways [20]. Another important cellular effect of corticosteroids is to exert an anti‐inflammatory and immunomodulatory action by stabilizing lysosomal membranes, suppressing prostaglandin synthesis, reducing histamine and bradykinin release, and capillary permeability [21]. Likewise, they reduce the number of inflammatory cells in various tissues by suppressing the production of chemotactic factors and the expression of cell surface molecules [22]. It is important to mention that the prolonged use of corticosteroids is associated with significant side effects related to the onset of metabolic disorders such as osteoporosis, hypertension, and diabetes. For this reason, alternative treatments have been implemented to reduce these side effects. Corticosteroids are commonly used across musculoskeletal disorders due to their strong anti‐inflammatory effects. They are applied in arthritis (osteoarthritis and rheumatoid arthritis), tendinopathies (rotator cuff, tennis elbow, and Achilles), bursitis (shoulder, hip, and elbow), spinal conditions (radiculopathy and stenosis via epidural injections), and soft tissue disorders such as plantar fasciitis and carpal tunnel syndrome. While they provide rapid pain relief, their benefits are short term, and repeated use carries risks like cartilage damage, tendon weakening, and systemic side effects [23].

Platelet‐rich plasma (PRP) is a blood‐derived product with platelet concentrations above baseline, rich in growth factors and cytokines that support tissue repair. It has gained attention as a promising, cost‐effective therapy for musculoskeletal pain and is already applied in several medical specialties, including orthopedics, cardiac surgery, and plastic surgery. Corticosteroid injections are widely recognized as the standard of care for managing sacroiliac joint pain in patients with chronic low back pain [24–27].

PRP exerts multiple biological effects at the cellular and tissue levels, stimulating the growth and differentiation of chondrocytes and osteoblasts, promoting mitogenesis in fibroblasts and smooth muscle cells, and regulating collagen synthesis while attracting macrophages and neutrophils [28, 29]. It also modulates key physiological processes, including angiogenesis, vascular permeability, bone formation, and cartilage regeneration, and plays a crucial role in mesenchymal cell division. These properties have led to its application in diverse fields, including dermatology for alopecia, gynecology for Asherman’s syndrome and ovarian insufficiency, orthopedics for musculoskeletal injuries, and neuroscience for nerve regeneration [30–32]. The literature consistently describes PRP as a source of accelerated tissue healing, with benefits such as enhanced bone regeneration, reduced inflammation, decreased blood loss in surgery, and faster wound healing. Moreover, PRP has been shown to alleviate pain in common conditions like osteoarthritis and tendon injuries, and in SI, its growth factors help restore anatomical function in degenerative contexts, offering a promising therapeutic alternative [33, 34].

It is timely to find effective and safe treatments for the management of chronic low back pain secondary to SI, given the high prevalence of this condition and its socioeconomic impact [35].

Identifying treatments that are effective and safe in the long term is crucial for improving patients’ quality of life and reducing the economic burden associated with this condition. Although corticosteroids are highly effective in the short term, they present significant risks with prolonged use [36]. The search for alternatives such as PRP is justified by the need for treatment options that offer sustained benefits over time without the adverse effects associated with corticosteroids. Furthermore, evidence on the use of PRP in the management of musculoskeletal pain has grown significantly in recent years [37], demonstrating the relevance of comparing these two treatment options for low back pain secondary to SI. Chronic and disabling diseases have been on the rise [38], and access to specialized treatments may be limited. It is important to consider the effectiveness of cost‐efficient and innovative treatments, such as the use of PRP.

This systematic review aims to provide a comparative evaluation of PRP injections versus corticosteroid injections for the treatment of low back pain secondary to SI. The goal is to support the adaptation of chronic low back pain management strategies to better suit local needs and contexts.

## 2. Methodology

This systematic review was structured according to the guidelines of the Preferred Reporting Items for Systematic Reviews and Meta‐Analyses (PRISMA) (see Table 1). We conducted a comprehensive electronic search of PubMed, Scopus, Web of Science, Cochrane Library, Google Scholar, LILACS, ScienceDirect, and ClinicalKey to identify studies published before 2024. Searches were performed on 24 June 2025. To enhance transparency and reproducibility, the full search strategy for PubMed is provided in the Supporting Information (Supporting Table 1). Searches combined controlled vocabulary (MeSH) and free‐text terms for PRP and sacroiliac joint conditions, together with terms for corticosteroids and injection‐based interventions. No language restrictions were applied. Search results were imported into Rayyan.ai, and duplicates were removed using the same reference manager, followed by manual verification. The PRISMA 2020 guidelines were followed, and the completed PRISMA checklist is provided in the Supporting Material. Once the potentially eligible articles were selected, a comparative table was created where the main characteristics of each article were presented using the following criteria: title, authors, publication date, study type, sample, type of intervention, additional interventions performed, and outcomes. In this way, 4 definitive articles were chosen (Table 2). One additional retrospective study [43] was identified during the selection process but was excluded from the primary analysis due to its retrospective design, which limits the ability to infer causal relationships.

**TABLE 1:** Preferred Reporting Items for Systematic Reviews and Meta‐analyses (PRISMA) statement to synthesize the results found in exploratory systematic reviews.


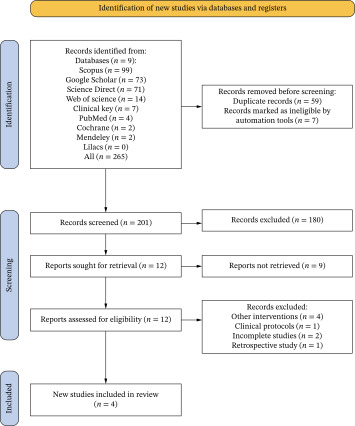

**TABLE 2:** General characteristics of the selected articles.

Title of the article	Authors	Year	Place	Type of study	Analyzed parameters	Intervention	Method of administration	Conclusions	Reference
Intra‐articular platelet‐rich plasma vs. corticosteroid injection for sacroiliac joint pain: a double‐blinded, randomized clinical trial	Chen et al.	2022	Columbia University Medical Center and Weill Cornell Medical Center	Randomized double‐blind controlled trial	Pain score (NPRS) and Oswestry disability index (ODI) over time	(*n* = 11) 1 mL of betamethasone sodium phosphate and acetate suspension (6 mg/mL) plus 1 mL of 2% lidocaine vs. (*n* = 15) 2 mL of PRP	Fluoroscopy‐guided intra‐articular injection	The steroid injection and PRP groups showed improvements in pain and function over time. The steroid group had significantly greater improvements in pain and function compared with the PRP group at 1, 3, and 6 months.	[39]
Ultrasound‐guided injection of steroids versus rich plasma for sacroiliac joint chronic pain management	Mohamed et al.	2024	The main university hospital of Alexandria, Egypt	Randomized Controlled Trial	Provocation tests, pain score (VAS), Modified Oswestry Disability Questionnaire (MODQ), patient satisfaction (SAPS)	(*n* = 25) Methylprednisolone 40 mg/mL and bupivacaine (0.5% and 0.25%) vs (*n* = 25) 2 mL of PRP, 0.5 mL of calcium gluconate, bupivacaine (0.5% and 0.25%)	Ultrasound‐guided intra‐articular and periarticular injection	The provocation tests, VAS score, and MODQ score in the steroid group showed greater short‐term improvement, but the PRP group had better long‐term outcomes. There were no significant differences in patient satisfaction or complications between the two groups.	[40]
Comparison of the efficacy of intra‐articular platelet‐rich plasma with intra‐articular steroid in the management of pain due to sacroiliac joint dysfunction	Dev et al.	2023	Tertiary care hospital in northeastern India	Prospective, randomized, parallel‐group study	Pain improvement	(*n* = 25) Autologous PRP (1–2.5 mL) vs. (*n* = 25) 40 mg of triamcinolone diluted to 1–2.5 mL	Fluoroscopy‐guided intra‐articular injection	PRP is superior to corticosteroid injection, showing better long‐term pain improvement and reducing the need for rescue analgesics or reinterventions at 6 months.	[41]
Evaluation of platelet‐rich plasma vs. steroid in the treatment of sacroiliitis by ultrasound‐guided injection in patients with seronegative axial spondyloarthropathies	Soliman et al.	2021	Department of Physical Medicine, Rheumatology, and Rehabilitation, Menoufia University Hospitals, from 2017 to 2019	Randomized, interventional, and comparative clinical trial	Pain improvement, functionality, disease activity with the (BADSAI) scale, physical function (BASFAI), and structural changes assessed through MRI	(*n* = 35) 1 mL of triamcinolone acetonide (40 mg) and 1 mL of 2% lidocaine vs. (*n* = 35) 2–3 mL of PRP and 1 mL of 2% lidocaine.	Ultrasound‐guided sacroiliac injection	PRP is a safe and effective technique for treating sacroiliitis in seronegative spondyloarthritis (SPA) and can completely improve active sacroiliitis, as shown by magnetic resonance imaging. Long‐term follow‐up and larger sample sizes are needed to assess the lasting effect of PRP on chronic sacroiliitis in SPA patients.	[42]

Supporting Table 1: PubMed search strategy: This table provides the electronic search strategy used for the PubMed database, in accordance with PRISMA 2020 guidelines. Equivalent strategies were adapted for other databases (Scopus, Web of Science, Cochrane, etc.) using corresponding field tags.

## 3. Results

An exhaustive search in multiple databases resulted in the identification of 265 potentially relevant articles. After eliminating 59 duplicate articles, the titles of the remaining 206 articles were reviewed, with 180 of them being discarded for not meeting the preliminary criteria. Subsequently, the titles and abstracts of the remaining 21 articles were evaluated, selecting 12 for a full‐text review. Applying strict inclusion and exclusion criteria, 4 articles were finally chosen that met all the requirements to be considered in this review. The general characteristics of the chosen articles are presented in Table 2. The risk of bias of the included randomized controlled trials was assessed using the Cochrane risk of bias tool, and the results are presented in Table 3.

**TABLE 3:** Risk of bias for randomized controlled trials using the standardized Cochrane tool.

Intention‐to‐treat	Unique ID	Study ID	Experimental	Comparator	Outcome	Weight	D1	D2	D3	D4	D5	Overall
Yes	CHEN2022‐PAIN	Chen et al., 2022	PRP intra‐articular injection	Corticosteroid (betamethasone)	Pain (NPRS)	1	Some concerns	Low risk	Low risk	Low risk	Some concerns	Some concerns
Yes	MOHAMED2024‐RAIC	Mohamed et al., 2024	PRP injection	Corticosteroid	Pain (VAS)	1	Low risk	Low risk	Low risk	Low risk	Some concerns	Some concerns
Yes	ULUSOY2023‐MEDS	Ulusoy et al., 2023	PRP (2 mL)	Corticosteroid	Pain (VAS)	1	High risk	Low risk	Low risk	Low risk	Some concerns	High risk
Yes	DEV2023‐IJP	Dev et al., 2023	PRP (fluoroscopy‐guided)	Methylprednisolone	Pain (VAS)	1	Some concerns	Low risk	Low risk	Low risk	Some concerns	Some concerns
Yes	LABEEB2021‐MMJ	Labeeb et al., 2021	PRP (US‐guided)	Triamcinolone	Pain (VAS)	1	Some concerns	Low risk	Low risk	Low risk	Some concerns	Some concerns

*Note:* D1: randomization process; D2: deviations from intended interventions; D3: missing outcome data; D4: measurement of outcome; D5: selection of reported result.

The randomized, double‐blind clinical study conducted by Chen et al. compared intra‐articular PRP injections with corticosteroid injections for the treatment of sacroiliac joint pain. A total of 26 patients met the inclusion criteria and were divided into two groups for each treatment. Regarding pain response, the corticosteroid group showed a significantly greater response compared with the PRP group at all time points (1, 3, and 6 months). The efficacy of PRP and betamethasone was primarily evaluated using the Numeric Pain Rating Scale (NPRS) and the Oswestry disability index (ODI) with follow‐up at 1, 3, and 6 months. Both groups showed improvements in function; however, if we objectively observe both interventions, regarding pain measured with the NPRS, both groups showed a significant reduction throughout the follow‐up period (*p* < 0.001 within‐group) although the magnitude of the improvement was greater in the corticosteroid group during the first 2 months. At 4 weeks, the betamethasone group reported a mean decrease of 4.2 ± 1.5 points, compared with 3.1 ± 1.4 points in the PRP group, a statistically significant difference (*F* (1, 22) = 7.767 and *p* = 0.011). This trend continued at 3 months, with persistently lower pain in the steroid group, although without reaching clinically relevant significance according to the minimum clinically important difference (MCID) criteria. Regarding the ODI, which assesses the functional impact of pain, both treatments showed significant improvement compared with baseline values (*p* < 0.05), but the differences between groups were not significant after the third month (*p* = 0.094), indicating convergence of functional outcomes in the medium term. At 6 months, patients treated with PRP showed a trend toward better functional performance (average ODI reduction of 28% versus 21% with corticosteroids) although this difference did not reach statistical significance, probably due to the small sample size (*n* = 24). It is emphasized that more studies are needed to fully understand the benefits and limitations of both therapies [39].

The randomized controlled clinical trial conducted by Mohamed et al. analyzed 50 patients, who were divided into two groups: Corticosteroid Group: methylprednisolone 40 mg/mL and PRP Group: 2 mL of PRP, applied via ultrasound‐guided intra‐articular and periarticular injection. Scales such as the visual analog scale (VAS), Modified Oswestry Disability Questionnaire (MODQ), and SAPD were used, with follow‐up visits at 2, 4, 6, and 8 weeks, as well as again at 4 months. This study compared measures using independent groups *t*‐tests, and it was shown in the short term (2–4 weeks) that the corticosteroid group had better results, measured by VAS (average decrease of 55% vs. 38% in PRP), with a statistically significant difference (*p* < 0.05). However, in the long term (8 weeks and 4 months), the PRP group had better results in these same measures; this group showed progressive and sustained improvement in both VAS and MODQ scores, achieving an average pain reduction of 72% and a functional improvement of 68%, while the corticosteroid group showed a rebound effect, with partial loss of analgesic response and mean improvement values below 45%. These differences were statistically significant in favor of PRP at 8 weeks and 4 months of follow‐up (*p* < 0.05). There were no significant differences between the groups in patient satisfaction (SAPS score) or overall efficacy and complications [40].

Dev et al. conducted a prospective randomized clinical study over 24 months. They included 50 patients randomly divided into two groups, receiving autologous PRP injections (Group A) or corticosteroid (40 mg triamcinolone) injections (Group B). The outcomes were measured using a numerical rating scale (NRS) that evaluates pain improvement on a scale of 1–10, and were measured before and after the procedure, at 24 h, at the first, third, and sixth months, in addition to recording the need for rescue analgesia injections or reintervention. The NRS before the intervention showed an average of 6.87 for Group A and 6.84 for Group B, with no significant differences. At 24 h, there were also no significant differences. However, Group A reported pain relief in 100% of the patients in the first month, 88% in the third month, and 76% in the sixth month. In Group B, only 20% and 28% reported relief at the third and sixth months, respectively. The *T* test was used as a statistical measure of all the above; the PRP group showed a gradual and sustained reduction in pain scores from baseline to 12 weeks (mean VAS: 7.8 ⟶ 2.8; *p* < 0.001), while the corticosteroid group improved initially (7.7 ⟶ 3.4; *p* < 0.001) but plateaued after 8 weeks. The difference between groups at 12 weeks was significant (*p* = 0.018), favoring PRP [44].

No patient in Group A needed additional interventions, while in Group B, four patients required NSAIDs, and one required a repeated corticosteroid injection at the fourth month. This research reveals that the use of PRP through a single intra‐articular injection surpasses corticosteroids in managing pain caused by sacroiliac joint dysfunction. Additionally, PRP not only proved to be safe but also provided superior results in terms of duration and efficacy of pain relief. With these findings, the study authors considered PRP to be a preferable therapeutic alternative for this condition.

An interventional study conducted by Labeeb et al. from 2017 to 2019 included a total of 70 patients, who were randomly assigned to two groups: Group I received triamcinolone (40 mg) and Group II received 2‐3 mL of PRP via ultrasound‐guided sacroiliac injection. Patients were evaluated with scales such as VAS, MODQ, and the spondyloarthritis activity and functionality indices (BASDAI and BASFI), before treatment, at 4 and 8 weeks postintervention. PRP injection resulted in improvements in the VAS for pain and the MODQ compared with corticosteroid injection. At 8 weeks, 68.6% of the PRP group had mild pain compared with 5.7% of the corticosteroid group (*p* < 0.001), and 74.3% of the PRP group had minimal disability compared with 14.3% of the corticosteroid group (*p* < 0.001). The study population consisted of patients with seronegative axial spondyloarthropathies, including ankylosing spondylitis, reactive arthritis, psoriatic arthritis, arthritis associated with chronic inflammatory bowel disease (Crohn’s disease and ulcerative colitis), and undifferentiated forms. At 8 weeks, Group II demonstrated significant improvement in both disease activity (BASDAI) and functional status (BASFI) following PRP treatment, whereas Group I showed no comparable benefit. A reduction in pain, evaluated by the VAS and MODQ scales of ≥ 50% at 4 weeks was observed in Group I, but not until 8 weeks in Group II, indicating a longer‐lasting effect with PRP. In contrast to the statistical measures employed, *T* and chi‐square tests were used to compare groups and proportions with a *p* < 0.001 regarding the improvement in functionality and disability in the PRP group [41].

## 4. Discussion

Low back pain secondary to SI is a condition that affects a considerable number of people worldwide, impacting the quality of life of those who suffer from it and increasing healthcare costs. As it is considered a chronic condition, various treatments alleviate symptoms in the short term but have adverse effects that are not equivalent to the benefits. This is why exploring unconventional treatments such as PRP injection in the sacroiliac joint has been a controversial topic that has shown positive results compared with conventional management with corticosteroid injections or oral analgesics.

The four clinical studies included in this review provide important data on the use of PRP and corticosteroid injections in the sacroiliac joint for the management of low back pain. The four studies analyzed the efficacy of both methods in different patient cohorts and follow‐up over time, allowing the identification of the best method in the short and long term. It was determined that the use of corticosteroids represents a significant improvement in low back pain secondary to SI between the second and fourth week of application [39, 40]. On the other hand, studies have shown that the use of PRP in this condition yields better long‐term outcomes. Authors reported follow‐up periods ranging from 8 weeks to 6 months after the application [41]. Additionally, it is highlighted that the group treated with PRP did not need rescue analgesia doses at the fourth month, while the group treated with corticosteroids did [39]. Only the study conducted by Chen et al. demonstrated the absolute superiority of corticosteroids over PRP use; however, it should be noted that this study included the smallest sample size of all the studies included in this review (26 patients) and emphasized that the difference was not statistically significant [39].

A retrospective study by Ulusoy et al. although not included in the primary analysis due to its study design reported findings consistent with the overall trend observed in the prospective studies. In that analysis, corticosteroids showed greater short‐term pain reduction, whereas PRP demonstrated more sustained improvement at later follow‐up points. However, because retrospective designs are inherently limited in establishing causal relationships and may be subject to selection bias, these results should be interpreted cautiously [43].

Each study included a different intervention; among the corticosteroids administered were methylprednisolone [40], betamethasone [39], and triamcinolone [41, 44]. This suggests that, although they belong to the same pharmacological group, their pharmacokinetic properties differ. Additionally, the concentration and volume of PRP varied across applications, with administered doses of 1, 2, and 3 mL [40, 41, 44].

Furthermore, recent clinical evidence, such as the randomized study by Fargaly et al. (2025), highlights the potential advantages of biological therapies in achieving sustained pain control in sacroiliac joint dysfunction. In this study, platelet‐derived therapy produced greater immediate analgesic effects and was associated with more durable pain reduction over time. These findings suggest that platelet‐derived products may provide progressive and sustained improvement by promoting tissue healing and modulating the inflammatory response within the sacroiliac joint [42].

Moreover, emerging evidence also supports the potential of these biological therapies to provide more sustained clinical benefits, beyond the previously mentioned findings. A study conducted by Manchikanti and colleagues concluded that biologic interventions such as PRP and cell‐based therapies may also provide progressive improvements in function, compared with conventional injections. In fact, these data support the hypothesis that platelet‐derived products exert their therapeutic effect not only through anti‐inflammatory modulation but also by tissue repair, and beyond these benefits, it has the advantage that several doses can be administered (as rescue medication) without repercussions regarding the acquisition of adverse effects or deterioration of clinical status in the short term [45].

The results indicated a general trend: corticosteroids provided effective pain relief in a short time after administration, while PRP offered long‐term benefits, likely due to the biological interactions of growth factors from platelets that generate chemotaxis, angiogenesis, and cell cycle induction, properties that corticosteroids do not possess. It should be emphasized that, to date, no adverse effects have been reported from the use of PRP through this type of administration, while the use of corticosteroids triggers soft tissue atrophy and bone demineralization. However, PRP preparation must be a standardized process with good manufacturing practices to avoid infections, and provide an adequate number of platelets and thus growth factors and other proteins that ensure its success.

Safety in this review was defined as the absence of serious adverse events (e.g., infection, neurovascular injury, and systemic complications) and the presence of only minor, transient effects such as local pain or swelling. Among the included studies, Dev et al. and Labeeb et al. reported no significant adverse effects in the PRP groups, while Mohamed et al. found no differences in complications between PRP and corticosteroids. Chen et al. did not provide detailed adverse event tracking. Overall, PRP was consistently associated with minimal local reactions and no systemic complications, whereas corticosteroids carry well‐documented risks with repeated use, including osteoporosis, hypertension, diabetes, and soft tissue atrophy.

In this review, corticosteroids consistently provided short‐term pain relief within 2–4 weeks, whereas PRP demonstrated more sustained benefits beyond the short‐term window. For clarity, we define “long‐term” improvement as outcomes observed at ≥ 3 months of follow‐up. Within this threshold, PRP showed progressive and durable analgesic and functional gains up to 6 months, while corticosteroid effects diminished after the early weeks. Although these findings support PRP as a safer alternative for repeated or prolonged treatment, the evidence remains limited by small sample sizes, methodological heterogeneity, and relatively short follow‐up periods, underscoring the need for larger studies with standardized protocols and longer observation.

Despite the strengths, our study presents some limitations, such as the considerable clinical and methodological heterogeneity among the studies included in this review, including differences in the specific drug administered, injection volume, preparation techniques, follow‐up periods, outcome assessment methods, and patient populations. This variability presents a major challenge for quantitative comparisons across studies, rendering a formal meta‐analysis unfeasible. Nevertheless, the qualitative synthesis reveals a consistent trend suggesting that PRP tends to provide more sustained analgesic and functional benefits over time, despite the variations observed among study protocols.

It is recommended that future studies include larger sample sizes and adopt standardized protocols for the preparation and administration of both PRP and corticosteroids. This would enhance the objectivity of the results and facilitate broader clinical implementation. Likewise, no studies were found that conduct long‐term follow‐up of patients under PRP treatment to confirm or rule out possible adverse reactions to this intervention.

## 5. Conclusions

This systematic review compared the efficacy of PRP and corticosteroid injections in the treatment of chronic low back pain secondary to SI. The findings in the reviewed literature indicate that both interventions are effective. However, they have different temporal profiles. On the one hand, corticosteroids provide faster pain relief in the first 2–4 weeks, whereas PRP shows greater and more lasting benefits from the eighth week and up to 6 months postintervention. Additionally, PRP was associated with a lower need for additional interventions and rescue analgesia, demonstrating a sustained effect over time. Furthermore, no significant adverse effects were reported with PRP application, in contrast to the known and mentioned risks of prolonged corticosteroid use. The results of this work are of great importance to the field of medicine, particularly in the area of nononcological chronic pain management attributable to the musculoskeletal system and regenerative medicine, as they propose PRP as a viable and safer long‐term alternative to corticosteroids. These findings are important for patients who require repeated or prolonged treatment, increasing treatment options.

PRP is generally considered a safe therapeutic option for pain management due to its autologous origin, which minimizes immunogenic risks and eliminates concerns about disease transmission. Its composition, rich in growth factors and anti‐inflammatory cytokines, promotes tissue repair and modulates inflammation, offering a biologically favorable alternative to corticosteroids. Clinical studies have consistently reported minimal adverse effects, typically limited to transient local discomfort. However, it is important to note that most available studies involve small patient samples and relatively short follow‐up periods, which may limit the robustness and generalizability of safety conclusions. Despite these limitations, the current evidence supports PRP as a well‐tolerated intervention with a favorable safety profile in musculoskeletal pain treatment.

The heterogeneity observed across studies should not only be acknowledged as a limitation but also explored as a potential explanation for differing outcomes. Variations in the corticosteroid type and dose, PRP preparation methods (activation status, platelet concentration, and injection volume), injection technique (intra‐ vs. periarticular and ultrasound vs. fluoroscopic guidance), follow‐up duration, and patient populations (mechanical vs. inflammatory SI) may meaningfully influence efficacy. Future research should aim to standardize protocols for both corticosteroid and PRP administration, adopt consensus guidelines for PRP preparation, stratify patients by underlying pathology, and employ uniform outcome measures with longer follow‐up. Larger multicenter trials with systematic adverse event reporting would help reduce variability and allow more robust comparisons, ultimately clarifying which patient subgroups benefit most from each therapy.

The inclusion of only five studies in this review could limit the strength of the conclusions. We believe, however, that this review remains valuable by identifying gaps in the literature and guiding future research in this emerging field.

## Author Contributions

All authors contributed to the study conception and design, data analysis, and interpretation. All authors participated in writing and revising the manuscript.

## Funding

This work was supported by the Universidad de La Sabana, through the research project with code: MED247‐2018, titled “EVALUATION OF THE EFFICACY AND SAFETY OF THE USE OF PLATELET‐RICH PLASMA IN THE MANAGEMENT OF PRESSURE ULCERS, PILOT STUDY.”

## Disclosure

All authors have approved the final version of the manuscript.

## Ethics Statement

Ethical approval was not required for this study because it involves the analysis of data from previously published studies.

## Conflicts of Interest

The authors declare no conflicts of interest.

## Supporting Information

Additional supporting information can be found online in the Supporting Information section.

## Supporting information


**Supporting Information** The supporting information includes additional information to enhance the transparency and reproducibility of this systematic review. Supporting Table 1 provides the complete PubMed search strategy used to identify relevant studies. The PRISMA 2020 checklist is also included as supporting information to ensure adherence to reporting standards. All other tables (Tables 2 and 3) are included within the main manuscript.

## Data Availability

All data analyzed in this study are derived from previously published studies cited in the manuscript and are available within the article.
