# Supplementary material for: Efficacy of Platelet‐Rich Plasma Injections Versus Corticosteroid Injections in the Management of Chronic Low Back Pain Secondary to Sacroiliitis
Source: Pain Res Manag. 2026 Jun 22;2026:8179046. doi: 10.1155/prm/8179046 (PMC13286872; doi:10.1155/prm/8179046)
Supplement: Supplementary file 1 — Supporting Information The supporting information includes additional information to enhance the transparency and reproducibility of this systematic review. Supporting Table 1 provides the complete PubMed search strategy used to identify relevant studies. The PRISMA 2020 checklist is also included as supporting information to ensure adherence to reporting standards. All other tables (Tables 2 and 3) are included within the main manuscript. [file PRM-2026-8179046-s001.docx]

**Supplementary Table 1.** PubMed search strategy. Search strategy adapted for other databases using equivalent controlled vocabulary and syntax.

##

| Database | Search date | Search strategy | Filters applied | Notes |
| --- | --- | --- | --- | --- |
| PubMed (MEDLINE) | June 10, 2024 | ("platelet-rich plasma"[MeSH Terms] OR "platelet rich plasma"[Title/Abstract] OR "platelet-rich plasma"[Title/Abstract] OR PRP[Title/Abstract]) AND ("sacroiliitis"[MeSH Terms] OR sacroiliitis[Title/Abstract] OR "sacroiliac joint"[Title/Abstract] OR sacroiliac[Title/Abstract] OR "sacro-iliac"[Title/Abstract]) AND ("corticosteroids"[MeSH Terms] OR corticosteroid[Title/Abstract] OR corticosteroids[Title/Abstract] OR steroid[Title/Abstract] OR steroids[Title/Abstract]) | Humans; publication date before 2024; no systematic reviews; no language restrictions | Adapted for other databases (Scopus, Web of Science, Cochrane) using equivalent field tags |
